# How much of the incremental protocol does a lactate threshold need? Agreement between three-stage and five-stage testing across seven lactate-derived indices in swimmers

**DOI:** 10.1080/07853890.2026.2737006

**Published:** 2026-10-09

**Authors:** Yupeng Shen, Xinnuo Zou, Xiaotong Chen, Xiaobin Wu

**Affiliations:** ^a^ School of physical education and sports science, South China Normal University, Guangzhou, China; ^b^ Sports Academy, Guangzhou College of Commerce, Guangzhou, China; ^c^ School of Physical Education, Chengdu Sport University, Chengdu, China

**Keywords:** Lactate threshold, Exp-Dmax, incremental testing, swimming

## Abstract

**Background:**

Incremental lactate testing is widely used to characterize the lactate–velocity relationship in swimmers, but the highest-intensity stages increase testing burden and may be difficult to execute consistently.

**Objective:**

To determine whether a five-stage protocol can be reduced to three stages without materially changing seven lactate-derived velocity indices, and whether model complexity affects reconstruction.

**Methods:**

Ninety highly trained swimmers completed a 5 × 200 m freestyle incremental test at 80–100% of 200 m personal-best performance. Stage-4 and stage-5 lactate values were reconstructed from the first three stages using participant-specific linear extrapolation and a long short-term memory (LSTM) model. Seven lactate-derived indices were recalculated and compared with full-protocol values using limits of agreement, typical error, ICC(A,1) and equivalence testing.

**Results:**

Lactate reconstruction was inaccurate for both models, yet v@Exp-Dmax showed close agreement with the full protocol (bias −0.008 m·s^−1^; limits of agreement −0.023 to +0.007 m·s^−1^; ICC 0.988) and met the pre-specified equivalence criterion. The other six indices did not meet equivalence. LSTM provided no advantage over linear extrapolation, and reconstructed v@Exp-Dmax preserved the performance-association pattern observed with the full protocol.

**Conclusions:**

A three-stage protocol may support lower-burden longitudinal monitoring of v@Exp-Dmax, but protocol simplification is index-specific and does not establish physiological threshold validity.

## 1. Introduction

Competitive swimming performance depends on long-term systematic training and the integration of multiple physiological capacities, among which aerobic capacity is particularly important. At major competitions such as the Olympic Games and World Championships, events range from 50 m to 1500 m. Middle- and long-distance events (200–1500 m) derive approximately 60–95% of their energy from aerobic metabolism [1]. These demands underscore the importance of assessing aerobic capacity when planning training for middle- and long-distance swimmers.

Aerobic metabolism generates energy primarily through the oxidation of carbohydrates and fats when oxygen supply is adequate [2]. In competitive swimming, aerobic capacity contributes to the ability to sustain high velocities during middle- and long-distance events and to use available energy efficiently [3]. Lactate is continuously produced and oxidized both at rest and during exercise, and blood lactate concentration reflects the net balance between its appearance and its clearance rather than the accumulation of an anaerobic by-product [4]. During high-intensity exercise, increased glycolytic flux raises lactate appearance, and blood lactate concentration increases when appearance exceeds clearance. Changes in lactate production, clearance and oxidation are associated with metabolic adaptation [5]. In practice, blood lactate responses are therefore used to monitor training load, evaluate metabolic efficiency and inform pacing strategies. The velocity at which this change becomes apparent is widely used as an operational index for pacing and energy distribution strategies in middle- to long-distance swimming events; it is described here as a curve-derived quantity rather than as a marker of the upper limit of aerobic energy contribution. Existing studies have demonstrated that during submaximal exercise, lactate-related parameters exert a more significant influence on endurance performance than maximal oxygen uptake [6]. This finding has been validated across both well-trained athletes [7] and untrained adults [8].

Several methods are used to assess aerobic capacity and derive lactate-related indices in swimmers, including maximal oxygen uptake testing, tethered swimming tests [9], the T-30 test [10], the T-400 test [11], critical velocity testing [12], maximal lactate steady-state testing [13] and incremental load testing [14]. Incremental load testing is commonly used in swimmers because it characterizes blood lactate responses under sport-specific conditions and can inform individualized pacing strategies. In practical training settings, the 5 × 200 m incremental protocol is frequently implemented [15], and its use in swimming has been described in detail by several groups [16,17]. Lactate–velocity (*L*–*V*) curve modelling is widely used to characterize athletes’ metabolic responses. Common approaches include logarithmic transformation (Log-Log), fixed blood lactate concentrations (e.g. OBLA at 2.0 and 4.0 mmol·L^−1^), curve-fitting methods (e.g. Dmax and Exp-Dmax) and turn-point methods (LTP1 and LTP2) [18–20]. These approaches provide operational indices that can inform training-load prescription and performance assessment. The second lactate turn point (LTP2) is defined operationally as the point on the *L*–*V* curve at which blood lactate concentration undergoes a second pronounced acceleration [21]; its relationship to maximal lactate steady state has not been established in swimmers and is not assumed here. The Exp-Dmax method uses exponential regression and identifies the velocity at the point of maximal perpendicular distance between the fitted curve and the chord joining its endpoints. In female runners, Machado et al. [22] reported that an exponential-plus-constant model produced indices that correlated strongly with 10-km performance, were less sensitive to the initial test intensity, and predicted performance better than traditional polynomial models. Whether the reduced sensitivity of the Exp-Dmax method also extends to the terminal portion of the curve has not been tested.

These indices are not interchangeable. Fixed-concentration, curve-fitting and turn-point methods rest on different operational definitions, return numerically different velocities and agree only partially with one another and with maximal lactate steady state in swimmers [23–25]. Each is an operational construct defined by an algorithm applied to a set of measured points, and each therefore carries its own dependence on how those points were obtained. In what follows we use the explicit terms v@Exp-Dmax, v@LTP2 and so on, and reserve the unqualified term ‘lactate threshold’ for the general concept rather than for any measured quantity.

Although the incremental load test is widely used in swimming practice, several challenges persist in its application. First, the test requires continuous completion across multiple intensity levels, demanding highly precise load control; fatigue accumulation or deviations in movement technique may cause result instability. Second, blood lactate sampling after each intensity level renders the procedure cumbersome and time-consuming, while the invasive nature of sampling reduces athlete cooperation and compliance. Furthermore, the aquatic environment introduces additional obstacles to physiological data collection, limiting test efficiency. These costs fall disproportionately on the final two stages, which are the most demanding for the athlete and the least reliably executed. Consequently, identifying low-burden, high-efficiency alternative methods while ensuring assessment validity has become a primary research focus.

This creates an obvious question and an equally important objection. The question is whether the protocol can be shortened – that is, whether the *L*–*V* relationship established by the first three stages contains sufficient information to reproduce the lactate-derived velocities obtained from the full five-stage test. The objection is that the final two stages capture the most nonlinear portion of the relationship and may therefore contribute information that is particularly important for curve fitting. Both issues are empirical, and neither has been examined systematically in swimmers. Importantly, the answer may differ among indices: methods based on the entire fitted curve, endpoints or local changes in slope may depend differently on the high-intensity region. The present study therefore quantifies agreement between lactate-derived velocities obtained from truncated three-stage and full five-stage protocols across seven indices in the same swimmers. Because reconstructing stages 4 and 5 requires an extrapolation model, and because it is not obvious how much modelling sophistication such a task warrants, we compare a two-parameter linear extrapolation with a long short-term memory (LSTM) network. That comparison is treated as a methodological control rather than as the object of the study.

## 2. Participants and methods

### 2.1. Participants

Ninety highly trained competitive swimmers were recruited for this study. Each participant had achieved a 200 m freestyle time corresponding to at least 84% of the current world record for their respective sex [26]. Participant characteristics are summarized in Table 1.

**Table 1.** Basic characteristics of the participants.

Sex	Age (years)	Height (cm)	Body mass (kg)	200-m freestyle time (s)	% of world record (%)
Male (*N* = 50)	14.54 ± 0.61	175.89 ± 6.36	63.66 ± 6.65	122.11 ± 4.56	84% ± 3%
Female (*N* = 40)	13.28 ± 0.94	165.63 ± 6.69	51.60 ± 7.01	133.04 ± 5.67	85% ± 4%

Applying the participant classification framework of McKay et al. [26], this sample corresponds to Tier 3 (‘highly trained’); the terms ‘elite’ and ‘high-level’ are not used. The squad was heterogeneous in event orientation: 32 swimmers were middle-distance specialists (200–400 m) and 58 were long-distance specialists (800–1500 m). All were tested with the same freestyle incremental protocol regardless of their primary competitive event. Training history was 6.2 ± 2.8 years and in-water training volume 40.6 ± 10.2 km·week^−1^.

All 90 swimmers completed the incremental test and contributed to the agreement analyses. Official competition results within the specified window were available for 89 of them; one swimmer therefore contributes to the agreement analyses but not to the performance analyses reported in Section 3.6.

All participants were required to obtain adequate rest within 24 h prior to testing and to avoid strenuous exercise or formal competitions to help standardize pre-test physiological conditions. To guarantee the accuracy and reliability of the tests, strict inclusion criteria were applied: (i) participants had no history of cardiopulmonary disease or recent sports-related injuries and had not taken any medications potentially affecting exercise performance within 30 days before testing; (ii) all participants had prior experience with the 5 × 200 m incremental load test, were familiar with the testing environment and procedures, and possessed the necessary physical and psychological readiness to complete the test. Written informed consent was obtained from all participants and, where applicable, their parents or legal guardians before testing.

Biological maturation was not assessed: neither maturity offset nor Tanner staging was collected. Given a mean age of 13.3–14.5 years, this is a material limitation and is treated as such in Section 5. The study was approved by the Ethics Committee of the School of physical education and sports science, South China Normal University (Approval no. SCNU-SPT-2025-021), and electronic guardian consent was obtained for participants under 16 years of age.

### 2.2. Methods

#### 2.2.1. Experimental procedure

Testing was conducted in a standard 50 m swimming pool that complied with competition regulations. Water temperature was maintained at 26–28 °C. Before testing, all participants received detailed instructions and were required to follow the standardized procedures. Resting blood lactate concentration, height and body mass were then measured. Participants subsequently completed an individual 800–1200 m in-water warm-up before the incremental test.

#### 2.2.2. Individualized incremental L–V test

An individualized incremental *L*–*V* test was conducted according to a previously established protocol [27]. Participants completed a 5 × 200 m protocol at target velocities corresponding to 80%, 85%, 90%, 95% and 100% of their 200 m freestyle personal-best velocity. A fixed 60 s recovery interval separated consecutive stages, and all repetitions began from the starting blocks. Immediately after each repetition, fingertip blood samples were collected poolside to measure blood lactate concentration. Swimming times were recorded using a SEIKO S141 stopwatch (Seiko, Tokyo, Japan), and blood lactate concentration was analysed using an EKF BIOSEN C-Line analyser (EKF Diagnostics, Barleben, Germany). Verbal encouragement was provided during the final stage to promote maximal effort (Figure 1). Following the test, lactate-derived velocity indices were calculated for each participant as described in Section 2.2.3.

**Figure 1.**
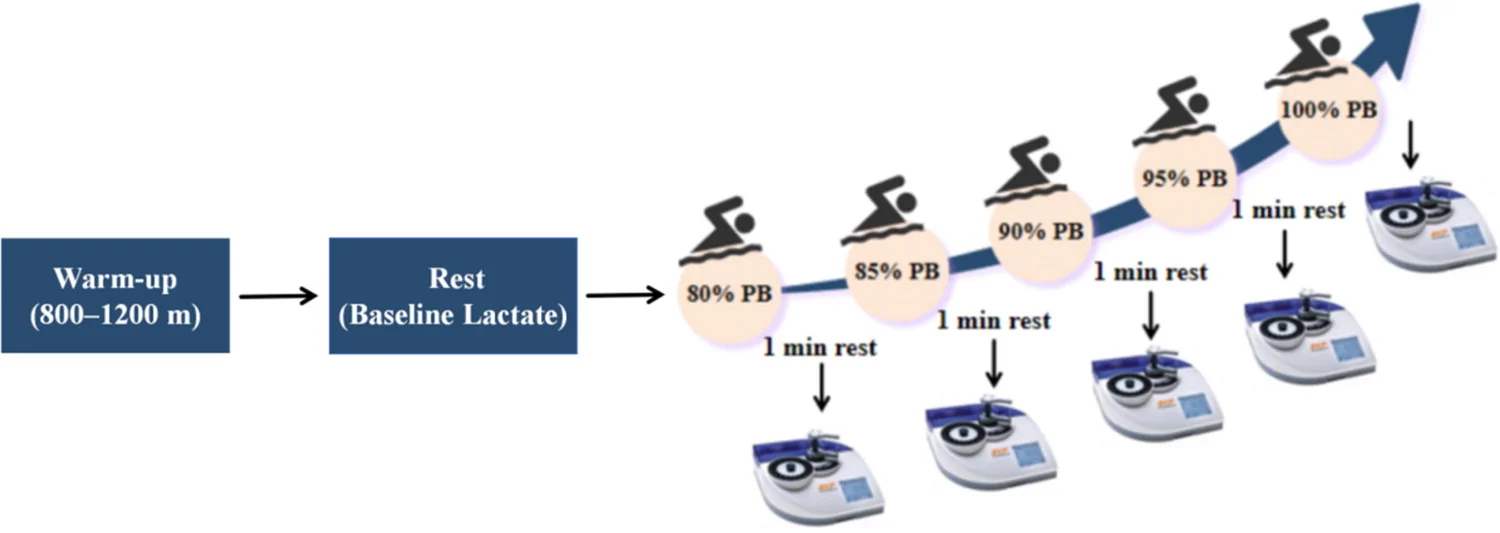
The flowchart of the 5 × 200 m incremental test.

Prescribing stages as percentages of 200 m personal best keeps relative intensity comparable across swimmers and is feasible at the poolside [28]. It carries a corresponding cost: absolute intensity at each stage depends on the swimmer’s 200 m freestyle ability, so swimmers oriented towards longer distances may find the early stages fall in a comparatively low metabolic zone. Furthermore, prescribed and achieved velocities are not identical; all analyses below use each swimmer’s achieved velocity at each stage rather than the prescribed value.

#### 2.2.3. Determination of lactate-derived indices

Lactate-derived velocities were computed with the lactater package in R, using swimming velocity as the intensity variable, the fitting form specified for each index and inclusion of the resting baseline point. Seven indices were obtained: Dmax, ModDmax, Exp-Dmax, Log-Poly-ModDmax, Log-Exp-ModDmax, LTP1 and LTP2. These names and abbreviations are used consistently throughout as operational curve-fitting outputs. The notation v@Exp-Dmax denotes the velocity at the point of maximum perpendicular distance between an exponential-plus-constant fit and the chord joining its endpoints [22]. The notation v@LTP2 denotes the velocity at the second marked acceleration of the *L*–*V* curve. Neither is presented as a physiological threshold; their relationships with maximal lactate steady state were not examined and are not claimed [29] (Figure 2).

**Figure 2.**
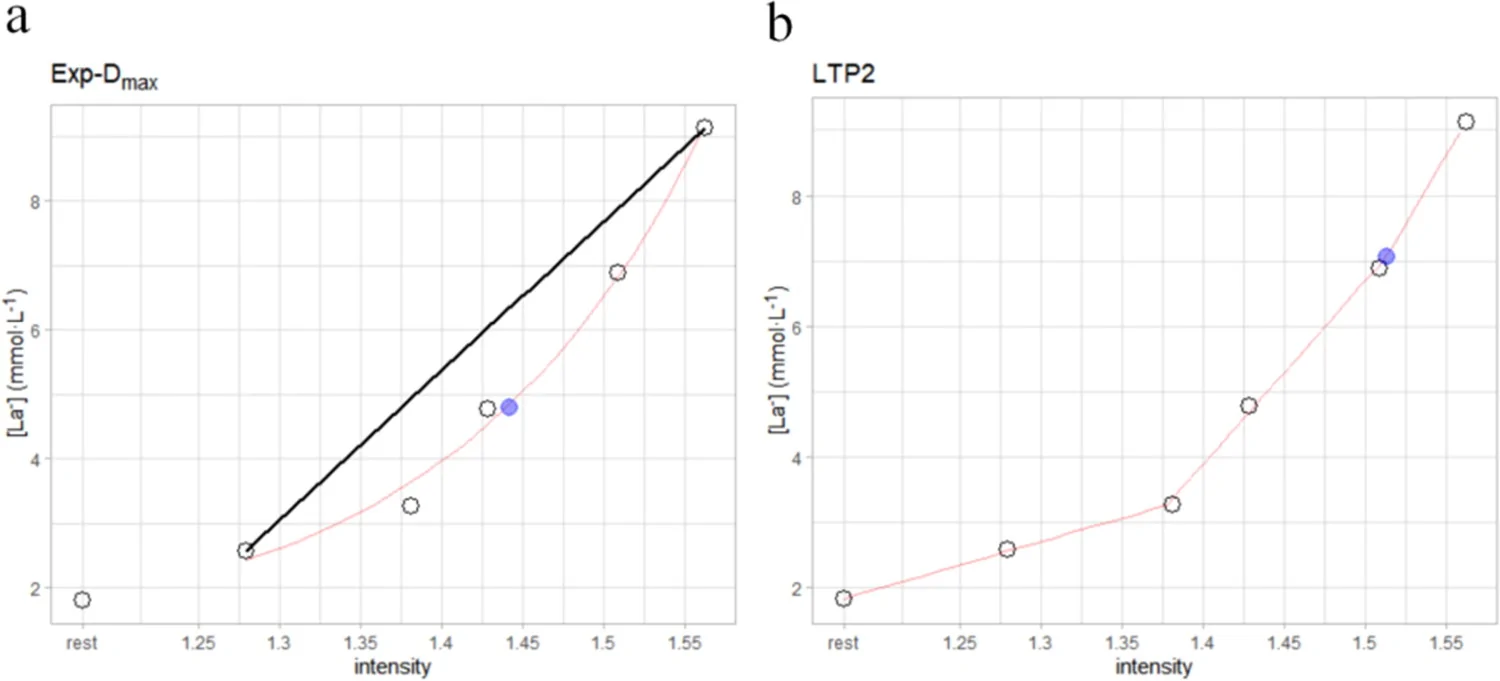
Illustrative diagrams of two lactate-derived indices: (a) Exp-Dmax and (b) LTP2, with the corresponding velocities highlighted.

#### 2.2.4. Performance data collection

Official 200–1500 m freestyle results obtained within 30 days before or after the incremental test were collected for each participant [30]. All performance data were taken from officially published competition result books. For each swimmer, the best result within this period was selected for analysis. Mean best race times were 126.90 ± 7.43 s for 200 m (*n* = 89), 264.70 ± 13.73 s for 400 m (*n* = 32), 538.37 ± 30.49 s for 800 m (*n* = 24) and 1001.14 ± 34.93 s for 1500 m (*n* = 17). These subsamples are not missing at random. Availability of an official result at a given distance depends on whether the swimmer contests that event, which in turn reflects event orientation: the 800 and 1500 m subsets are drawn from long-distance specialists and differ systematically from the full sample. This materially reduces the robustness of the performance analyses, both because the effective sample falls from 89 to as few as 17 and because the remaining swimmers are a selected group rather than a random subset. Estimates from these subsets are reported with confidence intervals throughout and are not read alongside the full-sample results.

#### 2.2.5. Reconstruction of stages 4 and 5

Two models were used to predict stage-4 and stage-5 lactate from each swimmer’s first three stages. Both were fitted separately for each participant; no parameters were shared across swimmers, and no information passed between them.

*Linear extrapolation*. For each swimmer, a two-parameter model (lactate = *a* + *b* × velocity) was fitted by ordinary least squares to their three stage-1 to stage-3 observations and evaluated at their achieved stage-4 and stage-5 velocities. No scaling or normalization was applied.

*LSTM*. For each swimmer, stage number and achieved velocity were used as input features and lactate as the target. Velocity and lactate were scaled to [0, 1] by min–max normalization fitted exclusively on stages 1–3; the inverse transformation likewise uses only stage 1–3 parameters, so no stage-4 or stage-5 information enters the prediction pipeline at any point. Inputs were reshaped to (1, 3, 2). The network comprised one LSTM layer (40 units) and a dense output layer, trained for 50 epochs with the Adam optimizer (learning rate 0.001) and mean squared error loss, implemented in Python (scikit-learn, TensorFlow). Hidden-unit count and time-step were selected in preliminary work on a single participant; this is reported explicitly and its limitations are addressed in Section 5. Because network initialization is stochastic, each participant’s model was fitted under five random seeds and predictions averaged; seed-to-seed dispersion is reported.

Because models are fitted within participants, there is no cross-participant training set and hence no train/test partition or subject-level cross-validation. Evaluation independence arises instead from the fact that stages 4 and 5 never contribute to fitting: the reported errors are out-of-sample by construction. For the same reason, cross-participant overfitting – and the regularization techniques that address it – do not apply here. The relevant risk is extrapolation uncertainty within each participant, addressed in Section 5.

#### 2.2.6. Model performance evaluation

The predictive performance of the LSTM and linear extrapolation models was evaluated using three indicators: root mean square error (RMSE), mean absolute error (MAE) and mean absolute percentage error (MAPE). RMSE reflects the overall error amplitude, MAE indicates the average error level, and MAPE quantifies the relative error. Lower values across all three indicators indicate better model performance. To further evaluate associations between measured and reconstructed lactate-derived velocities and competition performance, correlation analysis was used to compare the relationships between the measured and reconstructed lactate-derived velocities and performance outcomes in the 200, 400, 800 and 1500 m freestyle events. Among them, the measured v@Exp-Dmax and v@LTP2 were calculated using the measured lactate concentrations from all five stages of the incremental load test. In contrast, the predicted values were obtained by using the LSTM and linear extrapolation models to estimate lactate concentrations at the final two stages from the measured data at the first three stages. The Exp-Dmax and LTP2 methods were then applied to both the measured and reconstructed *L*–*V* curves.

### 2.3. Statistical analysis

All data are presented as mean ± standard deviation (*M* ± SD). Agreement between lactate-derived velocities from the truncated and full protocols was assessed using Bland–Altman analysis (bias and 95% limits of agreement), typical error of measurement (TEM = SD of differences/√2) and ICC(A,1) (two-way random, absolute agreement, single measurement). Equivalence was tested using two one-sided tests (TOSTs) against a smallest worthwhile change of 0.015 m·s^−1^, corresponding to approximately 1% of the mean lactate-derived velocity. This bound was derived from the race-to-race performance variability of 0.8–1.1% reported for Olympic-calibre swimmers [31] and was fixed before the agreement analyses. Sensitivity to terminal-point error was quantified by perturbing reconstructed stage-5 lactate by ±0.5, ±1.0 and ±1.5 mmol·L^−1^ and recomputing each lactate-derived index. Associations with competition performance were examined using partial correlations controlling for 200 m personal-best velocity and hierarchical regression, with 200 m personal-best velocity entered at step 1 and reconstructed v@Exp-Dmax at step 2. Sex was not entered into the primary analyses: models were built independently within each participant, and agreement was assessed through within-participant paired comparisons. Sex-specific moderation was not evaluated. The two extrapolation models were compared in the same participants using paired *t*-tests of absolute error in lactate-derived velocity. Because seven indices were compared, nominal *p* values were interpreted against a Bonferroni-corrected α of 0.007. Analyses were conducted in R (lactater) and Python; analysis code and derived data are available on request.

## 3. Results

### 3.1. Data characteristics and analysable sample

Of the 90 swimmers, curve fitting failed for one participant on the measured data and for three participants on reconstructed data, in every case because achieved velocity was not monotonically increasing across stages, which can prevent convergence of the fitting routine. The common analysable sample for the primary index was therefore *n* = 86. Two features of the raw data bear directly on the question at issue. Achieved velocity was not monotonically increasing in 17 of 90 swimmers, reflecting pacing execution error under a protocol prescribed from 200 m personal best. More importantly, blood lactate did not rise monotonically across the first three stages in 29 of 90 swimmers. For roughly one third of this sample, therefore, the early portion of the curve does not by itself indicate the direction the curve will take at higher intensities.

### 3.2. Accuracy of lactate reconstruction

Reconstruction of stage-4 and stage-5 lactate was not accurate (*n* = 180 predictions). Linear extrapolation gave a MAE of 2.01 mmol·L^−1^ with a systematic bias of −1.75 mmol·L^−1^; the LSTM gave 2.69 and −2.68 mmol·L^−1^. Both models therefore underestimated high-intensity lactate substantially, as expected when extrapolating a convex relationship. Across five random seeds, the standard deviation of LSTM MAE was 0.108 mmol·L^−1^ – an order of magnitude smaller than the gap between models.

### 3.3. Agreement of lactate-derived velocities

Mean v@Exp-Dmax was 1.421 m·s^−1^. Agreement differed clearly among indices (Figure 3(a)). For v@Exp-Dmax obtained using linear extrapolation (*n* = 86), bias was −0.008 m·s^−1^, with 95% limits of agreement of −0.023 to +0.007 m·s^−1^ (±1.07% of mean velocity), TEM of 0.0055 m·s^−1^ and ICC of 0.988; equivalence was established (TOST *p* = 1.3 × 10–12). For the LSTM, the corresponding values were −0.009 m·s^−1^, −0.027 to +0.009 m·s^−1^ (±1.26%), 0.0064 m·s^−1^, 0.985 and *p* = 3.2 × 10^−9^, respectively. Every other index failed equivalence: Log-Exp-ModDmax (bias −0.026 m·s^−1^, ±4.66%, ICC 0.856), ModDmax (+0.026, ±5.55%, 0.842), Dmax (+0.032, ±5.70%, 0.819), LTP1 (−0.013, ±5.80%, 0.867), LTP2 (−0.041, ±6.46%, 0.758) and Log-Poly-ModDmax (+0.019, ±8.36%, 0.732). The limits of agreement for v@LTP2 were six times wider than those for v@Exp-Dmax. Although the bias for v@Exp-Dmax was within the equivalence bounds, it differed significantly from zero (*t* = −8.2): the truncated protocol underestimated v@Exp-Dmax by approximately 0.6% (Figure 3(b,c)).

**Figure 3.**
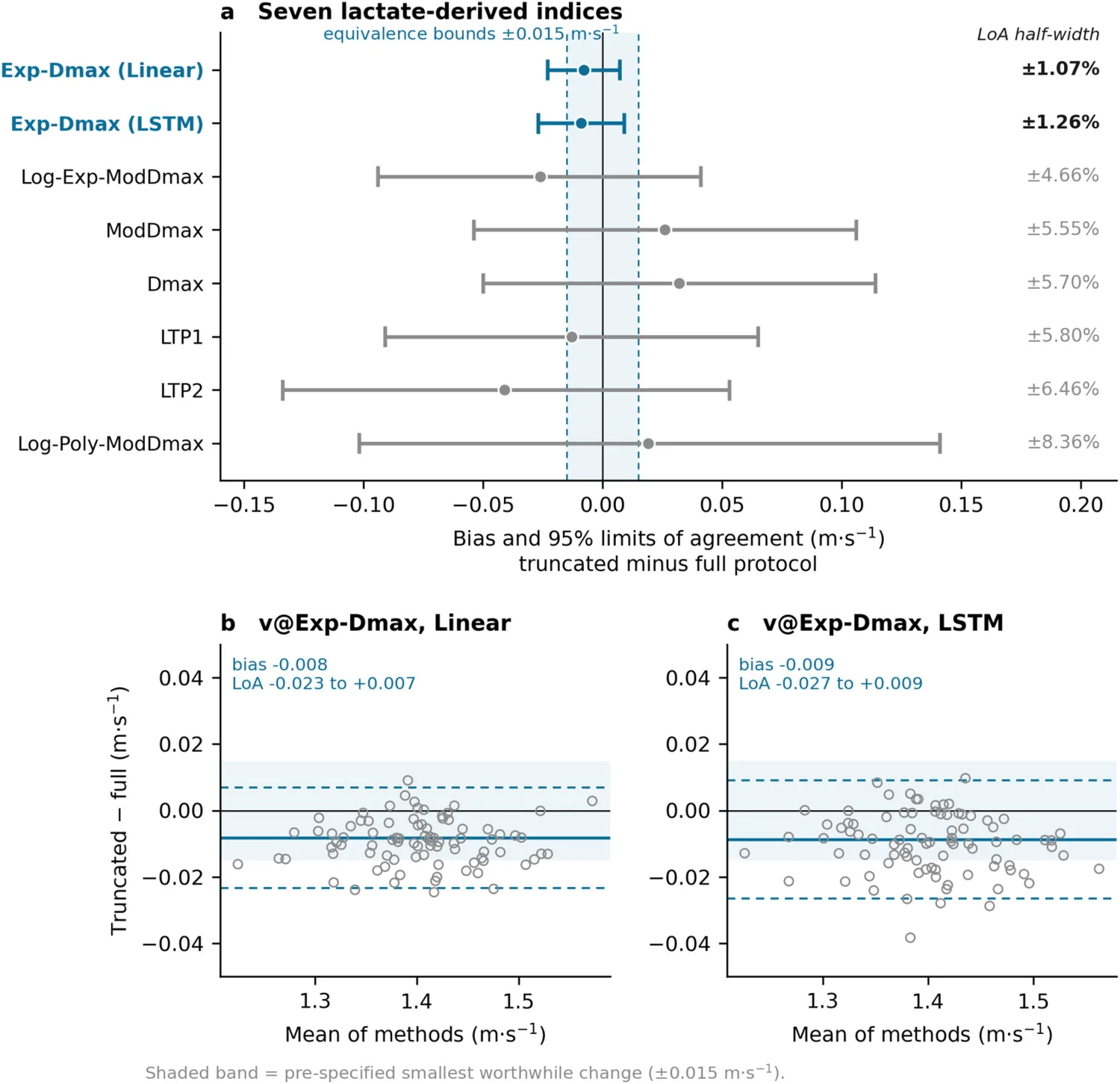
Agreement between three-stage and five-stage protocols. (a) Bias and 95% limits of agreement for seven lactate-derived indices; the shaded band marks the pre-specified equivalence bounds of ±0.015 m·s^−1^, and the right-hand column gives the limits of agreement as a percentage of mean lactate-derived velocity. (b, c) Bland–Altman plots for v@Exp-Dmax obtained using linear extrapolation and the LSTM.

### 3.4. Sensitivity to terminal-point error

Perturbing predicted stage-5 lactate shifted v@Exp-Dmax by approximately 0.002 m·s^−1^ per 1 mmol·L^−1^ (Figure 4(a,b)). This accounts for the apparent paradox between Sections 3.2 and 3.3: a systematic lactate underestimation of 1.75 mmol·L^−1^ translated into a v@Exp-Dmax bias of only about 0.008 m·s^−1^. Sensitivity was markedly greater for the other indices, whose individual shifts reached 0.10–0.27 m·s^−1^ under the same perturbations.

**Figure 4.**
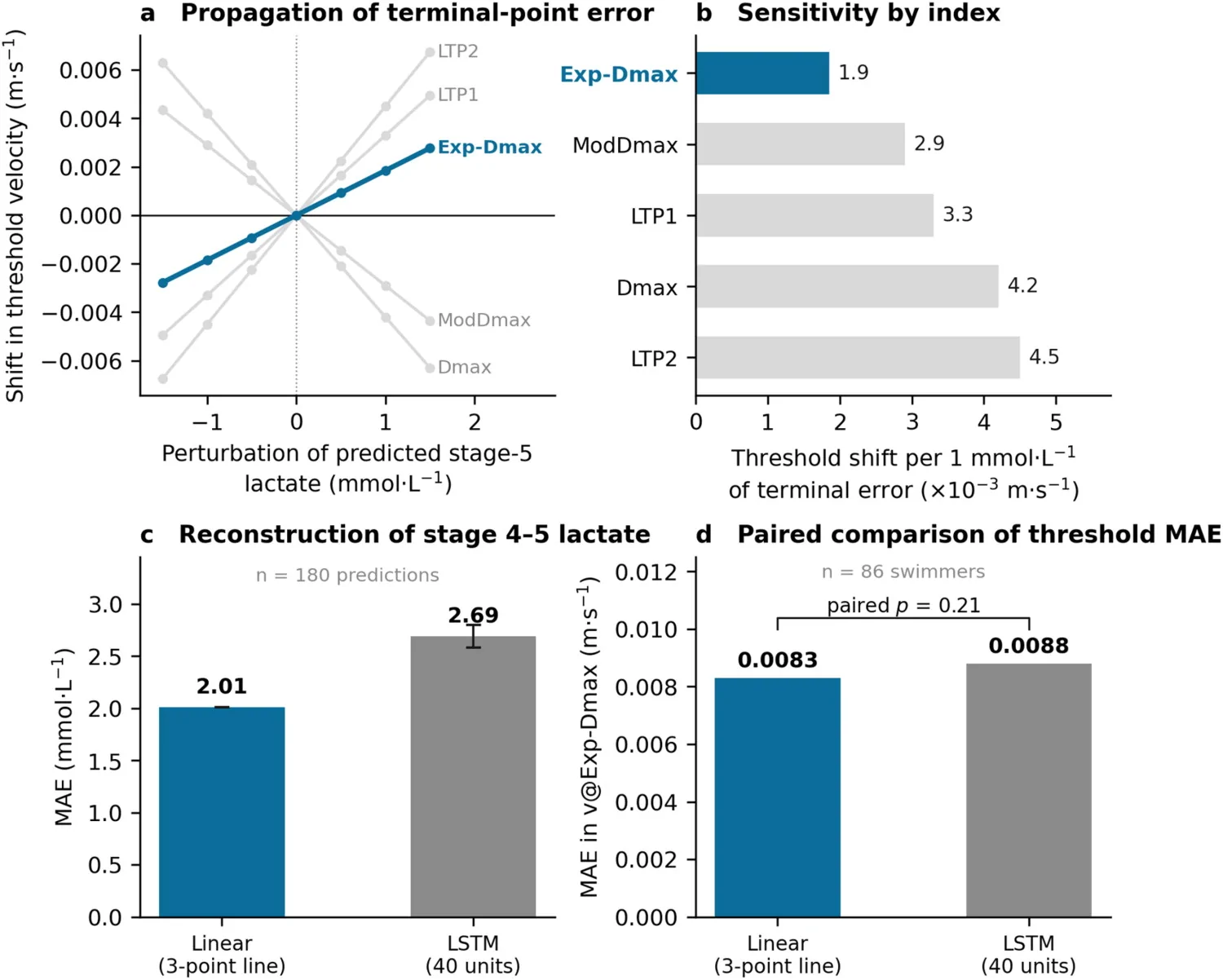
Robustness of Exp-Dmax and comparison of extrapolation models. (a) Shift in lactate-derived velocity when reconstructed stage-5 lactate was perturbed by ±0.5 to ±1.5 mmol·L^−1^. (b) Shift in each lactate-derived velocity per 1 mmol·L^−1^ of terminal error. (c) Mean absolute error in reconstructed stage-4 and stage-5 lactate; the error bar shows dispersion across five random seeds. (d) Mean absolute error in v@Exp-Dmax in a paired comparison of the same 86 swimmers.

### 3.5. Comparison of extrapolation models

For the primary index, the two models were indistinguishable: MAE in v@Exp-Dmax was 0.0083 m·s^−1^ for linear extrapolation and 0.0088 m·s^−1^ for the LSTM (paired difference 0.0005 m·s^−1^; *p* = 0.21; *n* = 86) (Figure 4(c,d)). The linear model produced marginally narrower limits of agreement and a marginally higher ICC. Two of the seven indices showed nominally significant differences (LTP1 favouring the linear model, *p* = 0.031; LTP2 favouring the LSTM, *p* = 0.042). These differences were in opposite directions, and neither remained significant after correction for seven comparisons; therefore, they were not interpreted as substantive.

### 3.6. Relationship with competition performance

Because every stage velocity is prescribed as 80–100% of 200 m personal-best time, v@Exp-Dmax is by construction close to a fixed fraction of 200 m personal-best velocity: across the full sample it corresponded to 89.2 ± 2.4% of v200PB, with *r* = 0.87 between the two. The zero-order correlation between reconstructed v@Exp-Dmax and 200 m race time was *r* = −0.88. This is reported as a consistency check, not as evidence of validity; a strong inverse association here is a necessary consequence of the protocol design.

After partialling out v200PB, reconstructed v@Exp-Dmax retained a moderate inverse association with 400 m performance (linear model: partial *r* = −0.404, 95% CI −0.663 to −0.058, *p* = 0.024, Δ*R*^2^ = 0.092; LSTM: *r* = −0.401, *p* = 0.025, Δ*R*^2^ = 0.090) and 800 m performance (linear: *r* = −0.652, 95% CI −0.839 to −0.329, *p* < 0.001, Δ*R*^2^ = 0.141; LSTM: *r* = −0.650, *p* = 0.001, Δ*R*^2^ = 0.146). For 800 m, this association was robust to leave-one-out deletion (partial *r* between −0.70 and −0.59). The decisive comparison was with measured v@Exp-Dmax: partial correlations obtained from the truncated protocol were essentially identical to those from the full protocol (400 m: −0.40 vs. −0.37; 800 m: −0.65 vs. −0.64). The truncated protocol therefore reproduced both the numerical value of v@Exp-Dmax and its association with competition performance.

The 1500 m subset (*n* = 16–17) yielded an effect of similar magnitude (partial *r* = −0.38 to −0.45) that did not reach significance. Because the confidence intervals spanned zero, this result was treated as inconclusive, and no inference was drawn. More generally, the performance analyses relied on progressively smaller and more selected subsets as race distance increased and were therefore less robust than the agreement analyses based on the full cohort. Expressing v@Exp-Dmax as a percentage of v200PB and correlating it with raw race times produced null results (all *p* > 0.23). This finding does not contradict the covariate-adjusted analyses because expressing v@Exp-Dmax as a percentage of v200PB is not equivalent to estimating the association between v@Exp-Dmax and race time conditional on v200PB.

## 4. Discussion

This study investigated the effectiveness of using a parameter-reduction approach to determine whether data from the first three stages of a five-stage incremental lactate test could reproduce lactate-derived swimming velocities obtained from the complete protocol. Using blood lactate concentrations from the first three stages together with the swimmers’ achieved velocities at stages 4 and 5, the LSTM and linear extrapolation models were used to reconstruct lactate concentrations at these two stages. Subsequently, seven lactate-derived indices were recalculated from the reconstructed curves and compared with those derived from the measured five-stage curves.

The principal finding was index-specific: v@Exp-Dmax showed close agreement with the full-protocol value and met the pre-specified equivalence criterion, whereas the other six indices did not. Importantly, the lactate concentrations at stages 4 and 5 were themselves reconstructed with substantial error. The agreement observed for v@Exp-Dmax therefore does not indicate accurate prediction of the omitted lactate concentrations, but rather reflects the relatively low sensitivity of this particular curve-fitting index to terminal lactate error.

Conventional methods for deriving lactate-related indices typically require data from at least five stages, and their outputs can be influenced by test design, execution quality and tolerance of high-intensity exercise [32]. Machine-learning approaches have also been applied to lactate-related outcomes: Erdogan et al. [33] used a multilayer perceptron neural network to predict heart rate at the onset of blood lactate accumulation (HR-OBLA) in soccer players; similarly, Etxegarai et al. [34] employed a recurrent neural network (RNN) in recreational runners to estimate a lactate-derived index. Considering that swimmers may have increasing difficulty maintaining prescribed pace and technical execution during the highest-intensity stages, and given the additional challenges of physiological data collection in aquatic environments, reducing the number of stages remains practically attractive.

However, the present findings show that such simplification cannot be generalized across lactate-derived indices. Exp-Dmax, other Dmax variants and turn-point methods are based on different operational definitions and therefore differ in their dependence on the high-intensity portion of the *L*–*V* curve [16,23–25]. The relative robustness of v@Exp-Dmax is consistent with its mathematical construction: changes in terminal lactate alter the fitted curve and its endpoint geometry, but the velocity corresponding to the maximal perpendicular distance may shift comparatively little on a smooth convex curve. In contrast, turn-point indices depend more directly on local changes in slope and were more sensitive to terminal error. Therefore, protocol reduction should be evaluated for the specific lactate-derived index being monitored rather than assumed to preserve a generic ‘lactate threshold.’ It should also be emphasized that the shortened protocol still requires three exercise stages and three fingertip blood samples; it therefore shortens rather than replaces conventional incremental lactate testing.

Although LSTMs are theoretically well suited to modelling nonlinear temporal sequences, the present analysis showed no practical advantage of the LSTM over the much simpler linear extrapolation model. This suggests that the current level of input information may be inadequate to leverage the strengths of deep learning models fully. Indeed, each participant contributed only three observations before extrapolation, providing very limited sequential information for a recurrent architecture and leaving a high-capacity model weakly constrained. The LSTM reconstructed lactate less accurately, while both approaches produced essentially equivalent errors for v@Exp-Dmax. For this specific application, the two-parameter linear extrapolation is therefore preferable because it is transparent, reproducible and readily implemented in practice. Rather than indicating a need for a more complex network, the present findings suggest that model complexity is not the principal limitation when physiological information is restricted to only three observations per individual.

Under the Exp-Dmax method, the reconstructed v@Exp-Dmax retained associations with competitive swimming performance, although these relationships require cautious interpretation. Because each stage was prescribed as a percentage of 200-m personal-best performance, v@Exp-Dmax was itself strongly related to v200PB. Consequently, the strong zero-order association with 200-m performance partly reflects the design of the protocol and should not be interpreted as independent evidence of validity. After controlling for v200PB, reconstructed v@Exp-Dmax remained moderately associated with 400-m and 800-m performance, and these associations were essentially indistinguishable from those obtained with the full five-stage protocol. The more informative finding is therefore that shortening the protocol preserved the association structure of the full-protocol output rather than that v@Exp-Dmax provides superior prediction of competitive performance.

The reliability of incremental lactate testing is influenced by the protocol, blood-sampling procedures and the athlete’s physiological condition on the day of testing [35,36]. In the present study, the limits of agreement for v@Exp-Dmax were approximately ±1% of mean swimming velocity, a magnitude similar to the race-to-race variability reported in highly trained swimmers [31]. This degree of agreement supports the potential use of the shortened protocol for longitudinal monitoring of an already established v@Exp-Dmax, but does not justify fine-grained individual training-zone prescription or athlete classification. A further practical boundary is that 29 of 90 swimmers did not show a monotonic rise in blood lactate across the first three stages. The early part of the curve may therefore be insufficient to establish an individual’s *L*–*V* profile for the first time. The shortened protocol is better viewed as a lower-burden option for repeated monitoring of an already established index. Finally, these findings concern agreement with a curve-fitting output and do not establish criterion validity against maximal lactate steady state or another independent physiological reference.

## 5. Limitations

(1) This study establishes agreement with a curve-fitting output rather than criterion validity against an independent physiological reference. Test–retest reliability of the full protocol and the longitudinal responsiveness of the truncated protocol were not directly assessed. (2) Generalizability is limited by the specific sample and protocol: participants were tier 3 swimmers aged 13–15 years [37,38], biological maturation was not assessed, and the findings require re-validation in other populations, event groups and sports. (3) Model and performance analyses were constrained by sparse individual data and selected subsamples. Each model was based on only three observations; some swimmers showed non-monotonic velocity or lactate responses, and race-performance analyses relied on small event-specific subsets, limiting initial profiling and broader performance inference.

## 6. Conclusions

The present study shows that reducing the burden of incremental lactate testing is primarily an index-selection problem rather than a model-complexity problem. For v@Exp-Dmax, a three-stage protocol combined with simple linear extrapolation offers a practical approach for longitudinal monitoring in highly trained adolescent swimmers, whereas the same simplification cannot be assumed to preserve other lactate-derived indices. Protocol reduction should therefore be validated for the specific operational index and intended application, and should not be interpreted as physiological threshold validation or as a substitute for criterion-based assessment.

## Data Availability

The datasets used and/or analysed during the current study are available from the corresponding author on reasonable request.
